# Illustrations

**Published:** 1873-06

**Authors:** 


### ILLUSTRATIONS.

A POOR ignorant colored man was once asked by an inquirer, how we could be in the Spirit and the Spirit in us? "O," replied the old man, "I put this poker in the fire and it gets red hot; it is in the fire, and the fire is in it. So the Holy Spirit can be in us and about us."

The doctrine of the Trinity—the three in one—has been illustrated by the fact that water exists in three different conditions, and for different uses in these conditions—water, ice, and vapor.

A clover stem has been found with three leaves, each distinct, yet making one stock.

So physiological results may be explained to the uninitiated, and may be thus brought within the comprehension of all. At the same time, it will not do to carry them out too minutely. For example: modern research seems to have established the fact that calomel does not act on the liver, and yet it has been received as a fact that it does so, for a century. But, even if it does not act directly on the liver, its being taken into the stomach is followed by a discharge of bile from the liver, into the intestines and outwards, the main fact remaining, that the effect of swallowing calomel is to procure an unloading of the liver of an excess of bile.

Half a century ago it was the favorite method of lecturers on physiology and anatomy in medical colleges, to say, of the process of digestion, that when anything was swallowed into the stomach, it went round and round, passing the discharging end, but that the valve, the gate-keeper, seemed to be possessed of positive knowledge, for, if a particle of food was presented and not fully digested, it would be refused permission to pass, and would be sent around again, like bits of ice stirred around with a spoon in a glass of water, if not wholly dissolved, give a few more turns and the work would be done. Still, if the undigested morsel was persistent, as a glass bead or a button, permission for exit would be given, as much as if the little gate-keeper was possessed of human intelligence, and would get out of patience, saying, "Come, old fellow, it's no use keeping you here any longer, out with you!" In this case, the one point of illustration sought to be impressed on the mind was this: that indigestible particles were kept in the stomach much longer than others.

"The heart beats ceaselessly," is not, literally, critically true, for it is at perfect rest for one-third of the time; yet the general fact is true, that it beats on and beats ever for a hundred years.

